# A risk stratification tool for prehospital triage of patients exposed to a whiplash trauma

**DOI:** 10.1371/journal.pone.0216694

**Published:** 2019-05-14

**Authors:** Artur Tenenbaum, Lena Nordeman, Katharina S. Sunnerhagen, Ronny Gunnarsson

**Affiliations:** 1 Hälsan & Arbetslivet, Occupational Health Care Unit, Region Västra Götaland, Alingsås, Sweden; 2 Department of Public Health and Community Medicine Institute of Medicine, The Sahlgrenska Academy, University of Gothenburg, Gothenburg, Sweden; 3 Research and Development Center Södra Älvsborg, Primary Health Care, Region Västra Götaland, Gothenburg, Sweden; 4 Institute of Neuroscience and Physiology, Department of Health and Rehabilitation, Unit of Physiotherapy, University of Gothenburg, Gothenburg, Sweden; 5 Institute of Clinical Neuroscience—Rehabilitation Medicine, University of Gothenburg, Gothenburg, Sweden; Cedars-Sinai Medical Center, UNITED STATES

## Abstract

**Objective:**

Our aim was to develop a risk stratification model to predict the presence of a potentially more sinister injury in patients exposed to a whiplash trauma.

**Methods:**

The study base comprised of 3,115 residents who first sought healthcare contact within one week after being exposed to a whiplash trauma between 1999–2008, from within a defined geographical area, Skaraborg County in south-western Sweden. Information about gender, age, time elapsed prior to seeking care, type of health care contact, and hospitalisation was retrieved. Seventeen potential risk factors were identified and evaluated using multivariable logistic regression.

**Results:**

Of 3,115 patients, 215 (6.9%) required hospital admission so theoretically 93% could have been initially assessed by primary health care. However, only 46% had their first contact in primary health care. All patients had symptoms resulting in a diagnosis of whiplash injury. Four risk factors were found to be associated with hospital admission: commotio cerebri (OR 31, 19–51), fracture / luxation (OR 11, 5.1–22), serious injury (OR 41, 8.0–210), and the patient sought care during the same day as the trauma (OR 5.9, 3.7–9.5). These four risk factors explained 27% of the variation for hospital admission and the area under curve (AUC) was 0.77 (0.74–0.80). Ninety-six percent of patients (2,985) had only a whiplash injury with no other injury. These could be split into those attending health care the same day as the trauma, 1,737 (56%) with a 7.1% risk for hospital admission, and those attending health care later, 1,248 (40%) with a 1.3% risk for hospital admission.

**Conclusion:**

Patients with no signs of commotio cerebri, no fracture/luxation injury, no serious injury, comprising 96% of all patients exposed to a whiplash trauma can initially be referred to primary health care for initial assessment. However, those contacting the health care the same day as the trauma should be referred to a hospital for evaluation if they can’t get an appointment with a general practitioner the same day.

## Introduction

Exposure to whiplash trauma due to a traffic accident is common and many seek health care. The initial clinical presentation varies but often consists of neck pain and other symptoms [1–5]. Up to 50% of those with symptoms after whiplash trauma, labelled whiplash associated disorders (WAD), face chronic health problems [6–13]. Reduced working ability occurs in 10–22% of individuals exposed to a whiplash trauma [14]. The annual costs of WAD are estimated to be 420 million euros in Sweden and 10 billion euros in Europe [15], most of it due to loss of production [16]. About 2.5% of the accidents leading to medical disability and insurance claims are from persons injured in modern cars that have whiplash protection [17]. Hence, this problem is unlikely to disappear soon and guidelines have been developed in several countries to facilitate initial management and rehabilitation to improve quality of life for patients with WAD [18–27]. Early clinical investigation, proper documentation, appropriate initial intervention, and rehabilitation are recommended to avoid chronicity [8, 10].

In Sweden half of individuals exposed to a whiplash trauma primarily attend a primary health care clinic [28], and in Australia it is around two thirds [29]. Some patients require a more thorough examination and imaging initially while others have a low risk for sinister injury. Patients at higher risk of sequel or death are better managed at hospitals [30] while the rest are better managed within primary health care. It is obvious that patients with visible severe injuries, severe comorbidities and incipient coma should be immediately referred to a hospital [30]. However, this is not a matter of black and white. It is a continuous gray scale between obviously severely injured patients and those with no obvious injury. Somewhere in the middle are individuals whose sole complaint is neck pain but they have no visible injuries. Should all of them be told to attend primary health care or should some of them be immediately referred to a hospital? This issue has not been clearly addressed before. The aim of this study was to develop a risk stratification model to predict severe injury after a whiplash trauma.

## Methods

This was a retrospective observational study using an accident and injury register in Skaraborg County, a part of the larger region of Västra Götaland in south-west Sweden. The county of Skaraborg in southern Sweden, which includes seven cities with a quarter of a million inhabitants, with hospitals and primary health care centers that all serve acutely injured people. The Regional Ethical Review Board of Gothenburg, Sweden approved the study (Registration number: 138–08, decision date 2008-04-28).

### The accident and injury register

The accident and injury register started up in 1997 and all healthcare facilities in this geographical area treating patients attending any health care facility after a trauma participated. The process of registering data in the database was as follows: the patient was asked for consent to be included in the register. If consent was obtained information about the trauma, provided by the patient or attending person was entered into the database. The physician in charge documented the diagnosis according to ICD-10 and recorded treatment, including any hospital admission.

A patient’s first attendance at any healthcare facility related to the trauma was registered irrespective of delay after exposure to the trauma. To ensure all patients exposed to an accident were included, a primary check was made by the secretary typing out the medical records and a secondary check was performed by comparing the administrative file of all patients and the cashier’s book. Missing cases were checked and all visits to the clinics due to any type of injury were compared with register entries, showing that 80% of all presenting cases exposed to an accident were properly included into the database during the study period of 1999–2008. The process of registration has previously been described in detail [28, 31, 32].

All hospitals and healthcare facilities dealing with acute injuries coded and classified patients according to three systems–the Nordic Medicinalstatistisk Committee's (NOMESCO) classification, European Home and Leisure Accident Surveillance System (EHLASS), and ICD-10.

### Information retrieved

The register contained information about patient demographics, diagnosis, if the patient was admitted to a hospital and also information about the accident itself. The present study extracted nineteen variables deemed relevant from the database for all patients classified as having a whiplash injury (ICD 10: S13.4) during the period 1999–2008: hospital admission, age, gender, healthcare contact, seeking medical care day- or night time, seeking medical care weekdays or weekend, seeking medical care in summer or winter, time elapsed between trauma and seeking care, if it happened during leisure time or while at work, trauma in the same direction of travel or not, car accident or another type of trauma, if the patient was the driver or front seat passenger, use of a seat belt. Finally, any concomitant diagnoses were registered such as contusion, commotio cerebri, wounds, fracture or luxation and other serious injury.

### Selection of patients

Patients given a diagnosis of whiplash injury with the ICD 10 code S13.4., irrespective of injury mechanism, and who sought first healthcare contact within one week after trauma. Some of these patients also had other diagnoses as described below. The formal diagnoses is used as a surrogate as to what kind of symptoms the patient likely had before arriving at the health care facility.

### Whiplash

All patients included were classified after evaluation by a medical practitioner as having a whiplash injury with the ICD10 code S13.4. Some of these patients also had other concomitant diagnoses. We let this indicate that these patients likely showed symptoms and signs from the neck, such as pain or reduced cervical range of motion, even before arriving to the health care clinic.

### Contusion

For the purpose of this study patients were defined to have a contusion if they were diagnosed with any of the following ICD 10 codes: M545, S000, S0001, S003, S005, S008, S009, S034, S101, S107, S109, S136, S169, S200, S202, S204, S208, S233, S234, S290, S300, S301, S337, S400, S403, S434, S435, S437, S468, S500, S501, S508, S534, S600, S602, S635, S626, S700, S701, S799, S800, S801, S807, S808, S836, S900, S901, S903, S909, S934. These patients were likely to show signs of contusion easily spotted by a paramedic before arriving to a health care clinic.

### Classification of commotio cerebri

For the purpose of this study patients were defined to have a commotio cerebri if they were diagnosed with the ICD 10 code S060. The prevailing criteria to diagnose commotio cerebri, or mild traumatic brain injury (mTBI), at the time for this study were a score of 15 or less on the Glasgow Coma Scale (GCS), without amnesia or focal neurological symptoms [33]. The GCS is as follows *Eye opening*: spontaneous 4, to sound 3, with pain stimulus 2, none 1. *Verbal response*: orientated 5, confused 4, words 3, sounds 2, none 1. *Motor response*: Obey commands 6, localises to painful stimuli 5, withdrawal from painful stimuli 4, abnormal flexion to painful stimuli 3, extension to painful stimuli 2, no movements 1.

Commotio cerebri, also labelled mild traumatic brain injury, is a highly subjective diagnosis which is often diagnosed by using a series of surrogate marker symptoms/signs [34]. Acute symptoms are transient or current, unconsciousness and/or memory loss/amnesia. Common secondary symptoms are headache, fatigue, memory and concentration difficulties [35]. The initial acute symptoms should easily be picked up by paramedics or nurses providing health advice over the phone.

### Wound

For the purpose of this study patients were defined to have a wound if they were diagnosed with any of the following ICD 10 codes: S000, S003, S005, S008, S009, S012, S013, S014, S015, S018, S019, S444, T150. Presence of an open would should be easily spotted by paramedics, nurses and even by lay persons.

### Fracture or luxation

For the purpose of this study patients were defined to have a fracture or luxation if they were diagnosed with any of the following ICD 10 codes: K081, S022, S024, S025, S029, S031, S032, S223, S322, S420, S423, S424, S430, S524, S525, S623, S626, S821, S824, S826, S837. Most of these fractures are painful enough to be strongly suspected even before arriving to the health care clinic.

### Other serious injury

For the purpose of this study patients were defined to have another serious injury if they were diagnosed with any of the following ICD 10 codes: S023, S120, S141, S220, S270, S320.

Examples could be; serious facial or spinal fracture, spinal injuries or thoracic injures often with pneumothorax. These injuries should be easily spotted by a paramedic or nurse in pre-hospital triage.

### Statistical analysis

Hospital admission was used as a surrogate marker for a potentially sinister injury. Multivariable logistic regression was used to analyse the relationship between the dependent variable hospital admission, and the other 17 independent variables. The variable attending at primary health care or hospital was not included in the multivariable logistic regression.

The assumptions used were that each patient (each observation) is independent from each other, that categorical variables are mutually exclusive, the data set contains enough observations to provide an answer to our research question, and that independent variables do not significantly correlate to each other. The latter was tested by assessing multicollinearity by examining the Tolerance and the Variance Inflation Factor (VIF).

A sensitivity analysis was done on the multivariable regression identifying outliers with residuals outside 2.0, 2.5 and 3.0 standard deviations (SD) respectively and the consequences on results by removing outliers. Based on this analysis a decision is made on which multivariable model should proceed.

Independent variables with p<0.05 in the chosen multivariable model were put into a new similar multivariable logistic regression to obtain a final model with adjusted odds ratios. The Area Under curve (AUC), with 95% confidence interval, was estimated as a measure of internal validation for the final multivariable regression model. The loss of patients in the final model was compared with all included patients to investigate if the loss represents any systematic bias.

Based on this final model beta-coefficients will be used to create a lookup table or probability nomogram for hospital admission after whiplash trauma. It will be developed by ranking the predicted probabilities of all possible permutations of the final predictors.

The level of statistical significance was set to 0.05. The statistical software used was IBM SPSS windows version 25.

## Results

Between 1999 and 2008, 265,324 events were registered. 3,368 patients were given a diagnosis of whiplash injury and 3,115 patients sought first healthcare contact within one week after injury. The average age for these 3,115 patients was 33 years (SD 16) and median 30 (interquartile range 20–44 years) (Table 1). Two hundred and fifteen patients (6.9%) were admitted to hospital and one of them initially sought care at a primary health care center (Table 1).

**Table 1:** Description of included patients.

	All cases (N = 3,115)			Admitted to hospital (N = 215)	
	Analysed	Distribution (number)	(%)	Distribution (number)	(%)
***Demographic factors***					
Age (years) *	3,115	Mean 33 (SD 16) Median 30(20–44)	——	Mean 36 (SD 19) Median 31 (20–48)	——
Gender: Female / Male	3,115	1,588 / 1,527	51 / 49	105 / 110	49 / 51
***Circumstances of first contact***					
Primary health care / Hospital	3,115	1,440 / 1,675	46 / 54	1 / 214	0.46 / 100
Attending Daytime / Night	1,924	1,303 / 621	68 / 32	87 / 81	52 / 48
Attending weekdays / Weekend	3,115	2,328 / 787	75 / 25	146 / 69	68 / 32
Attending Summer / Winter	3,115	1,529 / 1,586	49 / 51	114 / 101	47 / 53
Days from trauma to attending health care*	3,115	Mean 0.89 (SD 1.4) Median 0.0 (0.0–1.0)	——	Mean 0.19 (SD 0.69) Median 0.0 (0.0–0.0)	——
Attending Same day as trauma / day 2–7	3,115	1,843 / 1,272	59 / 41	192 / 23	89 / 11
***Circumstances***					
Leisure time / Work related	3,079	2,123 / 956	69 / 31	153 / 53	74 / 26
Collision in the same direction of travel /Another direction of trauma	3,115	1,184 / 1931	38 / 62	24 / 191	11 / 89
Car accident / Another type of trauma	3,115	2,240 / 875	72 / 28	108 / 107	50 / 50
Driver/ Passenger front seat	2,250	1,799 / 451	80 / 20	107 / 25	81 / 19
Seat belt on / Seat belt not on	1,967	1,628 / 339	83 / 17	58 / 44	57 / 43
***Diagnosis***					
Only whiplash injury (WAD)	3,115	2,620	84	92	43
WAD + Contusion	3,115	374	12	60	28
WAD + Commotio cerebri	3,115	89	2.9	58	27
WAD + Wound	3,115	123	3.9	18	8.4
WAD + Fracture or luxation	3,115	41	1.3	21	9.8
WAD + Other serious injury	3,115	9	0.29	6	2.8

* Mean values (standard deviation) and median. (25^th^ - 75^th^ percentile).

Four independent variables were statically significant predictors of hospital admission: patients attending health care same day as trauma, having commotio cerebri, fracture or luxation or other serious injury (Table 2). These four variables combined explained roughly one quarter of the variation in the dependent variable (Naegelkirke R square 0.27). The AUC was 0.77 (0.74–0.80, p 2.9x10^-40^), Omnibus test 10x10−^74^. Hosmer and Lemeshow Test 0.017. There was no multicollinearity in the first comprehensive regression (Table 2) with tolerance < 1.0 and VIF = 1.0 for all independent variables.

**Table 2:** Potential risk factors for hospital admission–no outliers removed.

	Multivariable logistic regression (n = 1,033)		Multivariable logistic regression (n = 3,155)	
	P	Odds ratio	P	Odds ratio
***Demographic factors***				
Increased age (one decade)	0.11	1.2(0.96–1.5)		
Female gender	0.35	1.4 (0.70–2.7)		
***Circumstances of first contact***				
Attending at Night	0.095	1.8 (0.90–3.5)		
Attending at Weekend	0.12	1.8 (0.84–4.0)		
Attending in Summer	0.17	1.6 (0.80–3.3)		
Attending same day as trauma	0.0012	23 (3.5–150)	<0.001	5.9 (3.7–9.5)
***Circumstances***				
Work related	0.11	1.9 (0.87–4.4)		
Trauma not in the same direction of travel	0.22	1.7 (0.74–3.7)		
Car accident	0.88	0.92 (0.31–2.7)		
Being passenger in front seat	0.88	1.1 (0.42–2.7)		
Not using seat belt	0.19	1.9 (0.72–5.2)		
***Clinical diagnosis***				
Only whiplash injury (WAD)	0.95	1.1 (0.15–7.4)		
WAD + Contusion	0.11	4.5 (0.71–29)		
WAD + Commotio cerebri	<0.001	73 (9.3–570)	<0.001	31 (19–51)
WAD + Wound	0.14	2.5 (0.75–8.0)		
WAD + Fracture or luxation	0.010	7.4 (1.6–33)	<0.001	11 (5.1–22)
WAD + Other serious injury	0.012	30 (2.1–420)	<0.001	41 (8.0–210)

### Sensitivity analysis

Using a cut-off of individuals with residuals outside a SD of 2.0 in the multivariable analysis including 17 independent variables identified 27 individuals, a cut-off of 2.5 SD identified 12 and a cut-off of 3.0 SD identified 0 individuals respectively. A closer inspection of these individuals did not reveal any coding errors. Removing individuals >2.5 SD (S1 Table) or >2.0 SD (S2 Table) added a few more variables statistically significant but the four significant variables identified before removing individuals remained highly significant with the highest odds ratios.

**Fig 1:**
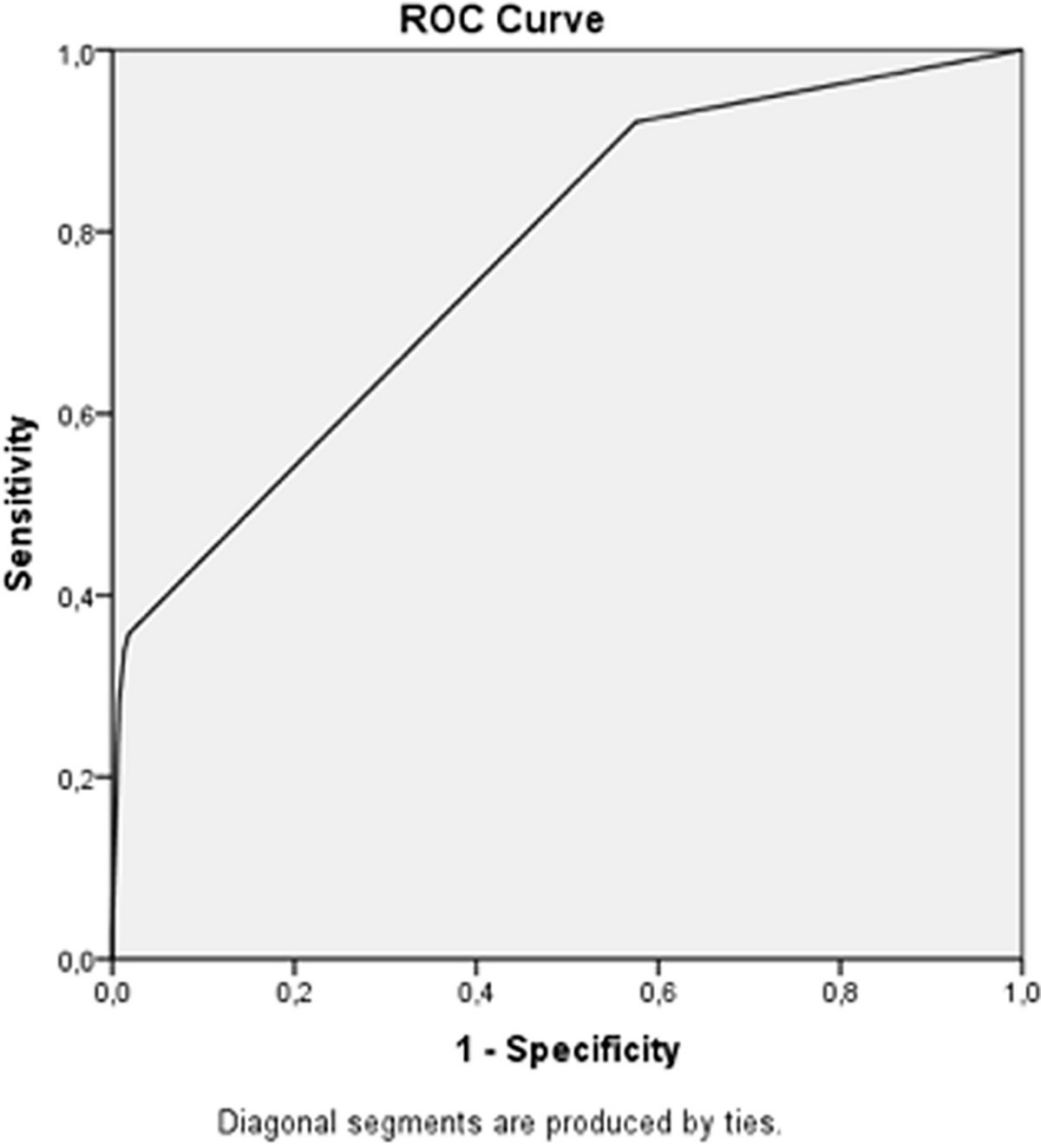
ROC Curve AUC 0.77 (074–0.80).

The final regression model, with no outliers removed, including 3,115 patients was used to create a lookup table predicting the need for hospital admission after whiplash trauma (Table 3 and Fig 1).

**Table 3:** Lookup table predicting the probability for hospital admission after whiplash trauma.

Probability of hospital admission %	Attending same day as trauma	Serious injury	Fracture luxation	Commotio cerebri	Number (tot = 3115) % (95% CI for)
100	yes	yes	yes	yes	n = 0 0 (0–100)%
99	no	yes	yes	yes	n = 0 0 (0–100)%
99	yes	yes	no	yes	n = 0 0 (0–100)%
97	yes	yes	yes	no	n = 2 0.064 (0.018–0.23)%
96	yes	no	yes	yes	n = 5 0.16 (0.069–0.38)%)
94	no	yes	no	yes	n = 0 0 (0–100)%
84	no	yes	yes	no	n = 0 0 (0–100)%
80	no	no	yes	yes	n = 2 0.064 (0.018–0.23)%
75	yes	yes	no	no	n = 4 0.13 (0.050–0.33)%
70	yes	no	no	yes	n = 68 2.2 (1.7–2.8)%
45	yes	no	yes	no	n = 27 0.87 (0.60–1.3)%
34	no	yes	no	no	n = 3 0.096 (0.033–0.28)%
28	no	no	no	yes	n = 14 0.45 (0.27–0.75)%
12	no	no	yes	no	n = 5 0.16 (0.069–0.38)%
7.1	yes	no	no	no	n = 1,737 56 (54–58)%
1.3	no	no	no	no	n = 1,248 40 (38–42)%

Analysis of the 1,033 patients included in the first logistic regression model with all 17 independent variables showed they were slightly older, 32 versus 29 years, than the patients that could not be included due to missing data. However, they were comparable in gender (p = 0.14) and admission to hospital (p = 0.10). There were no missing cases in the final regression analysis only including the four independent variables.

## Discussion

This study used a high-quality database to produce a risk stratification model for patients exposed to a whiplash trauma. This model can potentially be used in pre-hospital triage.

### Triage of patients exposed to a whiplash injury

Most patients, 96%, turned out to have a low risk for hospital admission. These patients were characterized by having no signs of commotio cerebri, fracture/luxation or serious injury. These patients should be referred to primary health care for the initial management. However, those contacting the health care the same day as the trauma should be referred to a hospital emergency department for evaluation if they can’t get an appointment with a general practitioner the same day.

Having the first assessment at the right level of care is important for optimal utilization of healthcare resources. During the ten-year period between 1999–2008, only 6.9% of patients with a whiplash injury required hospital admission. However, 54% of patients initially sought care at a hospital, rather than visiting a primary healthcare facility. The prediction model presented in this study has the potential to increase the proportion of patients directed to primary health care from 46% to 96%. This increase in patient flow for medical visits to primary care should be manageable as it represents less than 0,1% of all visits to primary health care.

### Methodological strengths

The main strength in this study is that it is based on clinical encounters in the healthcare system and is not limited to data from an insurance company. Strengthening the data quality, all patients included were examined by the physician in charge, who documented and registered a diagnosis according to ICD-10 after taking a history and performing a clinical examination. The quality of this database was closely monitored with regular checks of data quality between 1998 and 2008, and data from this period is of very high quality. With an inclusion rate of 80% it can be considered that the database reflects real life activities of managing acute trauma. There were negligible changes in the population in Skaraborg over this 10-year period reducing this as a potential confounding factor.

The multivariable logistic regression was done robustly where independent variables were checked for multicollinearity. There were no missing cases in the final regression analysis. Sensitivity analysis did not identify any extreme outliers with residuals >3.0 SD. Outliers with residuals > 2.0 and 2.5 SD respectively made some difference to the result including a few more variables. However, four variables remained strongly statistically associated with the dependent variable hospital admission irrespective of keeping or removing outliers. In the absence of extreme outliers (> 3.0 SD) and with no obvious coding errors it was decided to keep all individuals in the final multivariable model.

The predictors identified in this study are perhaps not surprising. However, this is to our knowledge the first time these variables have been weighted together to create a simple and clinically useful look-up table (Table 3).

### Methodological weaknesses

One weakness is that there are no data regarding insurance claims or compensation enabling the creating of a prediction model for permanent injury. The study base, Skaraborg County, is not necessarily comparable with studies in cities with a population larger than 100,000 where traffic may be more intense.

The strong association between concomitant presence of commotio, fracture / luxation or other serious injuries and hospital admission is likely to eclipse any relationship that might exist between other independent variables and hospital admission, for example age. However, it makes sense to use the strongest predictors to build a prediction model and the addition of other independent variables such as age is likely to only make a marginal contribution to the overall prediction model.

### Conclusion

This study shows that most individuals with no signs of other injuries than a potential whiplash injury can be referred to primary health care for initial management. This comprises up to 96% of all individuals. However, those contacting the health care the same day as the trauma should be referred to a hospital for evaluation if they can’t get an appointment with a general practitioner the same day. It would be of value to externally validate these results in another setting.

## Supporting information

S1 Table Potential risk factors for hospital admission–outliers >2.5 SD removed. (DOCX)

S2 Table Potential risk factors for hospital admission–outliers >2.0 SD removed. (DOCX)
