# Supplementary material for: A risk stratification tool for prehospital triage of patients exposed to a whiplash trauma
Source: PLoS One. 2019 May 14;14(5):e0216694. doi: 10.1371/journal.pone.0216694 (PMC6516661; doi:10.1371/journal.pone.0216694)
Supplement: S1 Table — (DOCX) [file pone.0216694.s001.docx]

**S1 Table. Potential risk factors for hospital admission – outliers >2.5 SD removed**

|  | **Multivariable logistic regression (n=1,031)** | | **Multivariable logistic regression (n=3,103)** | |
| --- | --- | --- | --- | --- |
|  | **P** | **Odds ratio** | **P** | **Odds ratio** |
| ***Demographic factors*** |  |  |  |  |
| Increased age (one decade) | 0.0083 | 1.4 (1.1-1.9) | <0.001 | 1.3 (1.1-1.6) |
| Female gender | 0.90 | 1.0 (0.45-2.4) |  |  |
|  |  |  |  |  |
| ***Circumstances of first contact*** |  |  |  |  |
|  |  |  |  |  |
| Attending at Night | 0.062 | 2.2 (0.96-5.2) |  |  |
| Attending at Weekend | 0.029 | 3.0 (1.1-7.9) | 0.0071 | 2.2 (1.2-4.0) |
| Attending in Summer | 0.48 | 1.4 (0.56-3.4) |  |  |
| Attending same day as trauma | 0.0033 | 31 (3.1-300) | <0.001 | 11 (4.2-30) |
|  |  |  |  |  |
| ***Circumstances*** |  |  |  |  |
| Work related | 0.050 | 2.9 (1.0-8.5) | 0.95 | 1.0 (0.55-1.9) |
| Trauma not in the same direction of travel | 0.43 | 1.5 (0.53-4.5) |  |  |
| Car accident | 0.94 | 0.95 (0.27-3.3) |  |  |
| Being passenger in front seat | 0.54 | 1.4 (0.45-4.6) |  |  |
| Not using seat belt | 0.028 | 3.9 (1.2-13) | <0.001 | 2.6 (1.5-4.7) |
|  |  |  |  |  |
| ***Clinical diagnosis*** |  |  |  |  |
| Only whiplash injury (WAD) | 0.62 | 0.57 (0.064-5.1) |  |  |
| WAD + Contusion | 0.082 | 6.1 (0.79-48) |  |  |
| WAD + Commotio cerebri | <0.001 | 140 (13-1500) | <0.001 | 44 (19-100) |
| WAD + Wound | 0.038 | 4.1 (1.1-16) | 0.88 | 2.4 (0.88-6.9) |
| WAD + Fracture or luxation | 0.016 | 8.1 (1.5-44) | <0.001 | 11 (4.2-29) |
| WAD + Other serious injury | 0.0048 | 49 (3.3-730) | <0.001 | 36 (3.9-330) |
