# Supplementary material for: A risk stratification tool for prehospital triage of patients exposed to a whiplash trauma
Source: PLoS One. 2019 May 14;14(5):e0216694. doi: 10.1371/journal.pone.0216694 (PMC6516661; doi:10.1371/journal.pone.0216694)
Supplement: S2 Table — (DOCX) [file pone.0216694.s002.docx]

**S2 Table. Potential risk factors for hospital admission – outliers >2.0 SD removed**

|  | **Multivariable logistic regression (n=1,006)** | | **Multivariable logistic regression (n=3,088)** | |  |
| --- | --- | --- | --- | --- | --- |
|  | **P** | **Odds ratio** | **P** | **Odds ratio** |  |
| ***Demographic factors*** |  |  |  |  |  |
| Increased age (one decade) | 0.33 | 1.2 (0.81-1.9) |  |  |  |
| Female gender | 0.46 | 0.59 (0.15-2.4) |  |  |  |
|  |  |  |  |  |  |
| ***Circumstances of first contact*** |  |  |  |  |  |
|  |  |  |  |  |  |
| Attending at Night | 0.14 | 2.8 (0.72-11) |  |  |  |
| Attending at Weekend | 0.0072 | 15 (2.1-110) | 0.012 | 2.3 (1.2-4.5) |  |
| Attending in Summer | 0.072 | 4.2 (0.88-21) |  |  |  |
| Attending same day as trauma | 0.0048 | 150 (4.6-4700) | <0.001 | 8.4 (3.1-23) |  |
|  |  |  |  |  |  |
| ***Circumstances*** |  |  |  |  |  |
| Work related | 0.024 | 13 (1.4-110) | 0.98 | 0.99 (0.48-2.0) |  |
| Trauma not in the same direction of travel | 0.23 | 4.1 (0.40-42) |  |  |  |
| Car accident | 0.37 | 2.2 (0.38-13) |  |  |  |
| Being passenger in front seat | 0.86 | 1.2 (0.13-11) |  |  |  |
| Not using seat belt | 0.016 | 11 (1.6-81) | 0.0026 | 2.6 (1.4-4.9) |  |
|  |  |  |  |  |  |
| ***Clinical diagnosis*** |  |  |  |  |  |
| Only whiplash injury (WAD) | 0.52 | 0.34 (0.013-9.1) |  |  |  |
| WAD + Contusion | 0.086 | 10 (0.72-150) |  |  |  |
| WAD + Commotio cerebri | <0.001 | 2200 (50-99000) | <0.001 | 44 (19-102) |  |
| WAD + Wound | 0.37 | 10 (1.2-96) |  |  |  |
| WAD + Fracture or luxation | 0.0021 | 94 (5.2-1700) | <0.001 | 19 (7.4-50) |  |
| WAD + Other serious injury | <0.001 | 615 (19-20000) | 0.0020 | 30 (3.5-260) |  |
